# Split BiRNN for real-time activity recognition using radar and deep learning

**DOI:** 10.1038/s41598-022-08240-x

**Published:** 2022-05-06

**Authors:** Lorin Werthen-Brabants, Geethika Bhavanasi, Ivo Couckuyt, Tom Dhaene, Dirk Deschrijver

**Affiliations:** Ghent University, IDLab - imec, 9000 Ghent, Belgium

**Keywords:** Computer science, Mathematics and computing

## Abstract

Radar systems can be used to perform human activity recognition in a privacy preserving manner. This can be achieved by using Deep Neural Networks, which are able to effectively process the complex radar data. Often these networks are large and do not scale well when processing a large amount of radar streams at once, for example when monitoring multiple rooms in a hospital. This work presents a framework that splits the processing of data in two parts. First, a forward Recurrent Neural Network (RNN) calculation is performed on an on-premise device (usually close to the radar sensor) which already gives a prediction of what activity is performed, and can be used for time-sensitive use-cases. Next, a part of the calculation and the prediction is sent to a more capable off-premise machine (most likely in the cloud or a data center) where a backward RNN calculation is performed that improves the previous prediction sent by the on-premise device. This enables fast notifications to staff if troublesome activities occur (such as falling) by the on-premise device, while the off-premise device captures activities missed or misclassified by the on-premise device.

## Introduction

Real-time activity recognition in a hospital room or nursing home is important, because it can help to detect troublesome events, such as the fall of a patient, as soon as possible. This is most meaningful for geriatric patients^1,2^ that are more likely to suffer lasting injuries from a fall, especially if treatment is delayed. To combat this, staff could set up cost-effective video cameras or other surveillance methods, however this is too privacy invasive. Another possibility is to provide the patient with a wearable device that can detect when a fall occurs, but this requires the patient to wear this device at all time, which may be forgotten or lost. This is where compact and cost-effective radar devices can alleviate some issues. Firstly, they are privacy preserving as no directly interpretable image can be extracted from the radar data. Secondly, the signals can penetrate walls and *see* through other materials^3^, allowing for monitoring in places where regular video equipment would not be installed for surveillance (e.g., a bathroom). Finally, in low light conditions, these systems also outperform traditional video-based surveillance systems.

Radar systems transmit electromagnetic radio signals that will be reflected by objects. If these objects are moving, the frequency of the signal shifts. This phenomenon is known as the Doppler effect. The reflected signal arrives back at the radar system after a certain amount of time from which the distance, angle of the object in relation to the sensor and speed can be derived. Using the superpositions of the reflections from different moving parts of a human body, a Micro-Doppler (MD) signature^4^ can be generated. This yields a vector of values containing speeds of objects moving to and from the radar.

In this paper a novel scalable model is presented to perform indoor human activity recognition, assigning an activity to every recorded time step. Its novelty lies in the decoupling of Bidirectional Recurrent Neural Networks (BiRNNs). A two-staged model, designed to work on separate devices with distinct Micro-Doppler streams, facilitates live monitoring of human activities in many different environments simultaneously, for example in a hospital with many rooms. The two-stage design is as follows:

1. An edge device computes the class predictions on a stream of incoming radar frames, using a lightweight model. This means results can be streamed in real-time.
2. Another more capable device (able to process large amounts of data in a batch) applies a backward model on intermediate computations made by the edge devices and improves the predictions. Any inaccurate predictions made by the edge device are rectified by this more capable device.

A key benefit of decoupling a BiRNN is that the hidden state of a regular RNN is used to supply a sliding window with information of past data, maximally avoiding redundant computations. As well as avoiding redundant computations, this technique also reduces the bandwidth needed between the edge and the cloud, as the precomputation done at the edge could reduce the amount of data needed to send to the cloud by half.

In “Related work” section, related work is discussed. In “Methodology” section, some mathematical foundations and the proposed model are explained. Different ways of evaluating the proposed method are explored in “Results” section, with “Conclusion” section containing concluding remarks.

## Related work

In literature, several solutions have been presented for activity recognition using the Micro-Doppler (MD) effect. Chen et al.^5^ use a Sparse Representation Classifier (SRC)^6^ that transforms the training data into a sparse representation, and finds the best fitting linear combination when testing an unseen data point. Making use of MD signatures extracted from Wi-Fi signals, their classifier can detect six distinct actions that are similar to the ones considered in this work. SRC is not commonly used, and the most prevalent method used in human activity recognition using radar is deep learning classification. Jokanovic et al.^7^ make use of Deep Neural Networks and stacked autoencoders to detect activities on 3 s windows, showing superior results using Deep Neural Networks, as opposed to convential and PCA based methods. Many of these works^7–12^ require neural networks on a device where prediction is done on a buffer of data, rather than handling every timestep seperately. This ultimately increases the amount of work done by the edge device. Another drawback is that a regular sliding window method does not carry over information from one windowed inference to the next. Recurrent Sliding Windows^13^ feed the previous predictions as input to the next predictions. While this is an improvement over the regular sliding window, this might not carry enough information for correct classification. The method presented in this work makes use of multiple hidden states to carry information from the past into the future.

Bhavanasi et al.^14^ introduce the PARrad dataset and perform activity recognition on people in a hospital room setting. Two different models are evaluated on both Range–Doppler (RD) maps and MD signatures: a Random Forest^15^ and a Convolutional Neural Network. These models are applied to windows of 40 frames, being 3.7 s. If the duration of the activity exceeds the window length, inaccuracies can occur. Vandersmissen et al.^8^ make use of Long Short-Term Memory (LSTM) units in a deep neural network to recognize hand gestures and various sedentary activities. This is also a windowed method, fixed to 2 s or 30 frames.

The proposed Split BiRNN method improves on these techniques by classifying every single frame of a stream of Micro-Doppler signatures, instead of classifying sliding windows.

As shown by Carrara et al.^16^, Recurrent Neural Networks and its derivates (Long Short-Term Memory networks, Gated Recurrent Units, ...) can be used to perform real-time inference. The Online-LSTM proposed only depends on a previous hidden state, which is used to calculate the next hidden state. The Offline-LSTM proposed is analogous to a bidirectional LSTM, which is used for more accurate, but offline predictions. There is no way of rectifying previously made prediction as new data comes in, as the online and offline LSTM proposed do not work in tandem.

The internet of things (IoT) could also benefit from the technique presented in this work. Roy et al.^17^ make use of a bidirectional LSTM for intrusion detection. Wardana et al.^18^ use a hybrid CNN and LSTM model for use on an edge device, such as a Raspberry Pi. Both of these methods are a one stage method, and require the edge device to be powerful enough to handle the model deployed.

The two-stage Split BiRNN method allows the edge device to be cheap and low-powered, with a more expensive cloud device utilized for batch recomputation. This approach requires less bandwidth than sending all of the data at once.

## Methodology

In “Split BiRNN” section, the novel method *Split BiRNN* is introduced. This method is validated on radar data in conjunction with a 1D Convolutional feature extractor in “1D convolutional feature extractor for micro-Doppler signatures” section, exploiting the locality present in MD signatures. The resulting network used for evaluation in this work is presented in “Network structure” section.

### Many-to-many classification

In^14^, a single prediction is made over a window of time. This class of models is called *many-to-one* models, where the *many* corresponds to the multiple time steps fed into the model, and the *one* corresponds to the single class assigned to all these input frames. While feasible to deploy in a real life setting, this window needs to be tuned to always span the duration of a single activity, and makes the assumption that an activity is visible in a certain period of time. In some cases, however, this is not possible due to the lack of context information. An example is the situation where a person is stationary during the execution of an activity. The model would not be able to identify the activity due to a lack of a significant MD signature in this time window. On the other hand, *many-to-many* models make a prediction for every input frame over time. Farsad et al.^11^ make use of a *many-to-many* sliding Bidirectional LSTM (BiLSTM) to assign a class to every frame. A bidirectional recurrent neural network consists of two different recurrent blocks. One processes the input data forwards in time, while the other processes the input data backwards in time. The hidden states are then concatenated. A limitation of the sliding BiLSTM over the novel approach presented in this work is that it needs more computational power as it does not make use of a simple feed forward RNN to make base predictions.

The technique proposed in this work—Split BiRNN—is a many-to-many model, leveraging the per-step predictions made by an RNN rather than aggregating hidden features and assigning a single class over a range of timesteps. The many-to-many model is applied on MD signatures, which is a Fourier transformed representation of radar chirps, resulting in 128 bins per timestep. Every timestep has a corresponding class.

### Split BiRNN

A considerable limitation of previous methods is the lack of scalability on low cost, low power devices. Large facilities wishing to monitor a multitude of radar streams simultaneously might not be able to supply a high powered device able to process a large amount of frames using a bidirectional recurrent neural network. Instead, this work proposes to run a small, unidirectional recurrent neural network on a low power device, and make use of intermediate hidden states on another, higher power device, that processes a batch of intermediate computations periodically. This work introduces the Split Bidirectional Recurrent Neural Network, or *Split BiRNN*, a real-time many-to-many classifier that is able to both give immediate results at a low computational cost, and subsequently more accurate results at a higher computational cost. In this work, this novel method is applied to MD signatures over time, but could be applied to any time series where RNNs are useful.

**Figure 1:**
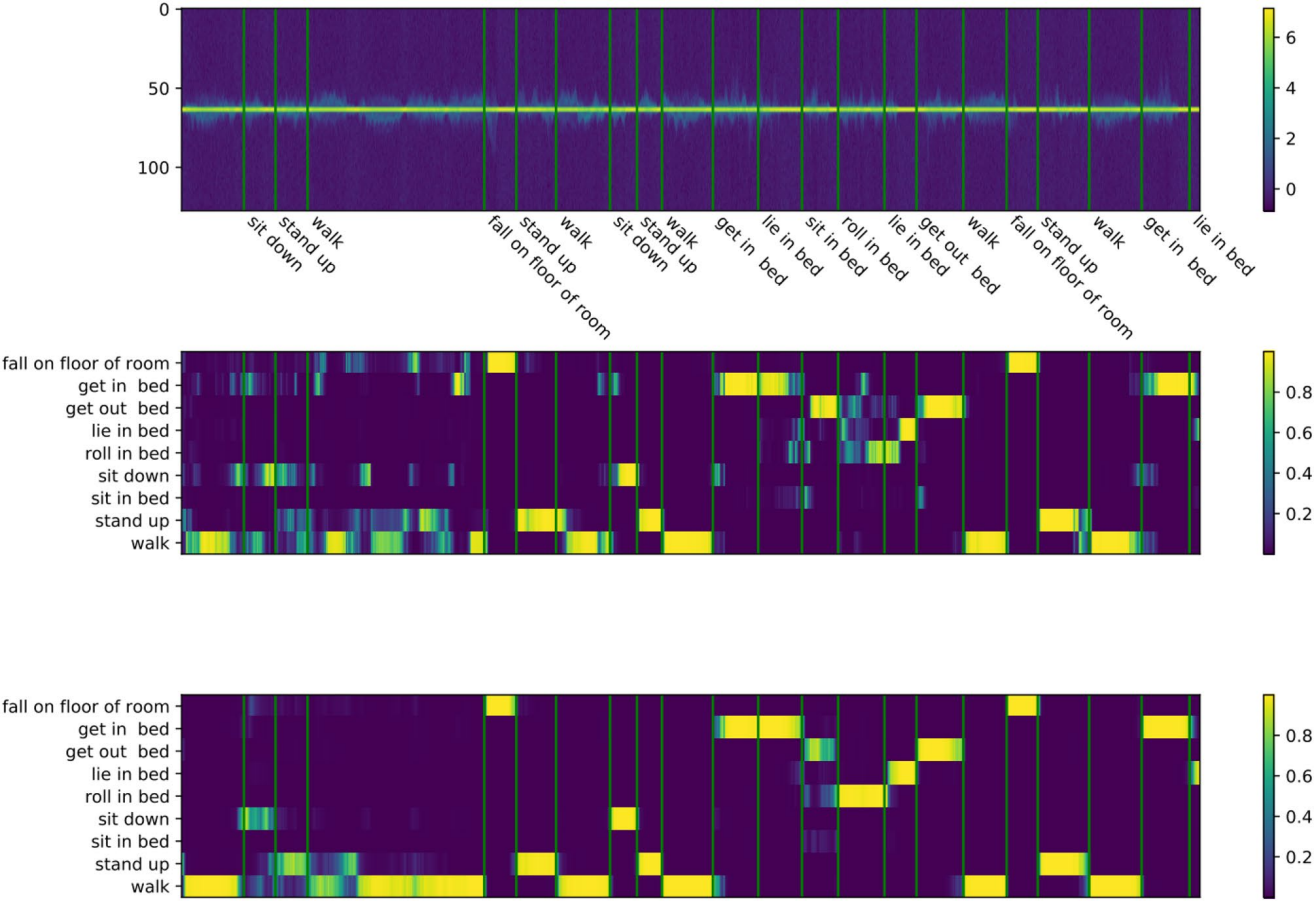
(Top) MD signatures over time in dB (middle) unidirectional RNN over time with classes on the *y* axis with activities annotated with vertical green lines. The color scale visualizes the confidence $\in [0, 1]$ of the model in a class. (Bottom) Bidirectional RNN over time with classes on the *y* axis. The color scale visualizes the confidence $\in [0, 1]$ of the model in a class.

To showcase the benefits of using a bidirectional RNN, a comparison of a strictly unidirectional network vs a bidirectional network applied to MD signatures over time is shown in Fig. 1. The unidirectional network resembles an event detector, and struggles to recognize sequences of similar activities accurately. The bidirectional network can incorporate more information into the predictions, hereby improving accuracy and decreasing noise.

The Split BiRNN technique consists of two linked networks called *forward branch* and *backward branch*. First, the forward branch is defined.

To update predictions as new data flows into the forward branch, the unidirectional nature of RNNs is used. These feed a state from one time step to the next, and only require the previous state and the current input to calculate the next state. The state of a regular RNN network can be described using the same notation as in^19^. The state of a forward RNN is denoted as $\overrightarrow {\mathbf {h}}^{(t)}$, with $t$ denoting the time step corresponding to this hidden state. $f$ is the non-linear function (LSTM^20^, GRU^23^, ...) applied to the input ${\mathbf {x}}^{(t)}$ at time $t$, parametrized by $\varvec{\theta }$ and $n$ refers to the corresponding layer.

1 $$\begin{aligned} \overrightarrow {\mathbf {h}}^{(t)}_{n+1} = f({\mathbf {x}}^{(t)}, \overrightarrow {\mathbf {h}}^{(t-1)}_{n}; \varvec{\theta }). \end{aligned}$$

This means that at time $t$, state ${\mathbf {h}}^{(t-1)}$ is the only state necessary to calculate state ${\mathbf {h}}^{(t)}$. This is different when making use of a Bidirectional RNN (BiRNN). In this case, an RNN layer is added that processes the input data in reverse. The resulting hidden states of the forward and backward RNN layers are then concatenated. This means the output is dependent on previous and following states when using a bidirectional RNN. The concatenation function between vectors is denoted as $+\!\!\!+$. Concatenation is used in this work to maximize the amount of data fed into the backward branch, but another addition operator could be used instead to save memory, or could be a learned operator. The backward RNN state is denoted as $\overleftarrow {\mathbf {h}}^{(t)}$. $\varvec{\theta _1}$ and $\varvec{\theta _2}$ are used to highlight the different parametrization between the forward and backward RNNs.

2 $$\begin{aligned} {\mathbf {h}}^{(t)}_n = f({\mathbf {h}}_{n-1}^{(t)}, \overrightarrow {\mathbf {h}}^{(t-1)}_n; \varvec{\theta _1}) +\!\!\!+f({\mathbf {h}}_{n-1}^{(t)}, \overleftarrow {\mathbf {h}}^{(t+1)}_n; \varvec{\theta _2}). \end{aligned}$$

In the presented model, the calculation of the forward and the backward state is split up as follows. The following is introduced, omitting the parameters $\varvec{\theta }$. For convenience, the general deep case is noted, with $\overrightarrow {\mathbf {h}}^{(t)}_0 = \overleftarrow {\mathbf {h}}^{(t)}_0 = {\mathbf {x}}^{(t)}$:

3 $$\begin{aligned} \overrightarrow {\mathbf {h}}^{(t)}_n&= f(\overrightarrow {\mathbf {h}}^{(t)}_{n-1}, \overrightarrow {\mathbf {h}}^{(t-1)}_n) \end{aligned}$$

4 $$\begin{aligned} \overleftarrow {\mathbf {h}}^{(t)}_n&= f(\overrightarrow {\mathbf {h}}^{(t)}_{n-1}+\!\!\!+\overleftarrow {\mathbf {h}}^{(t)}_{n-1}, \overleftarrow {\mathbf {h}}^{(t+1)}_n) \end{aligned}$$

5 $$\begin{aligned} {\mathbf {h}}^{(t)}_n&= \overrightarrow {\mathbf {h}}^{(t)}_n +\!\!\!+\overleftarrow {\mathbf {h}}^{(t)}_n. \end{aligned}$$

**Figure 2:**
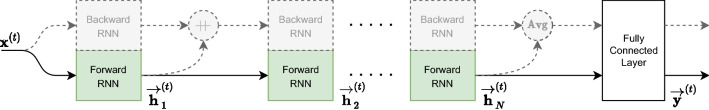
Graphical model of the forward step. The backward step is grayed out.

The forward branch of the network follows the regular structure of a deep RNN with a fully connected layer for predictions, as seen in Fig. 2.

The backward branch differs from regular deep BiRNN networks because it is run separately from the forward branch, but shares the same weights in the final fully connected layer. In a regular BiRNN, the output of the forward and the backward RNNs would be concatenated and fed into the next layer. The model differs in that the forward RNN always operates without knowledge of the output of the backward layer. This has the advantage that both branches can be jointly trained, reducing the full model size. This is not necessary, as a separate fully connected layer could be trained instead.

**Figure 3:**
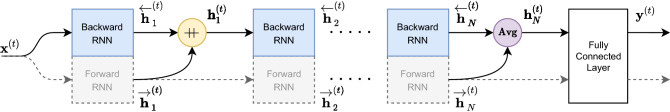
Graphical model of the backward step. The grayed out lines and blocks in the forward step indicate that this step is no longer necessary because the values are pre-computed in the forward step, and can be reused.

The states $\overrightarrow {\mathbf {h}}_n^{(t)}$ calculated in the forward branch are reused when calculating the backward branch. Figure 3 shows how they are concatenated, as described in (5).

Because the same Fully Connected layer is used in the forward branch as in the backward branch, the input to this layer needs to match the shape of the forward layer. Instead of using concatenation, the average is used. This has the added benefit of keeping the output activation values in the same space as in the forward branch. $N$ denotes the index of the last layer of the deep recurrent network.

6 $$\begin{aligned} {\mathbf {h}}_{N}^{(t)} = \frac{\overrightarrow {\mathbf {h}}_{N}^{(t)} + \overleftarrow {\mathbf {h}}_{N}^{(t)}}{2}. \end{aligned}$$

#### Real time implementation

The Split BiRNN conceptually has no benefits over the regular BiRNN concerning classification or regression accuracy, as the structure remains very similar. However, Split BiRNN can be deployed on different devices, so the forward branch can provide immediate predictions on one device, and the backward branch performing a second, more accurate pass on another device by way of sliding window.

In this scenario the forward branch runs continuously, updating its state every time step, while the backward branch runs on sliding windows of the last $L$ intermediate computations, with a certain hop size $H$. For an input ${\mathbf {X}} \in {\mathbb {R}}^{t \times d}$, a prediction is performed over frames ${\mathbf {x}}^{(t-L+1)},\dots ,{\mathbf {x}}^{t}$, every $H$ timesteps. The forward branch is applied per frame, and retains a hidden state over all of its computations. The forward branch loop is shown in Algorithm 1.
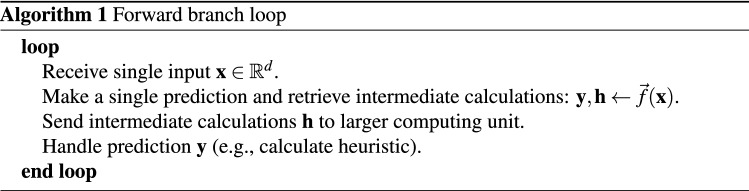


The backward branch loop requires a calculation buffer containing the last $L$ intermediate computations generated by the forward branch. Every $H$ frames, an improved prediction is computed, replacing the previous prediction made by the forward branch. This algorithm is shown in Algorithm 2.
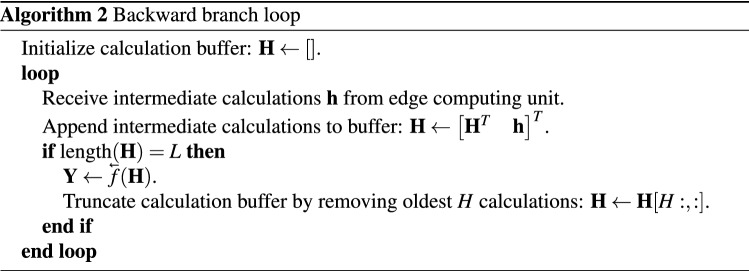


### 1D convolutional feature extractor for micro-Doppler signatures

While the Split BiRNN could be used on its own on Micro-Doppler signatures over time, it can not exploit the locality of single MD signatures. Therefore, a feature extraction step prior to the Split BiRNN is proposed when working with MD signatures over time, providing dimensionality reduction.

Convolutional Neural Networks (CNN) are one of the most popular and widely used types of neural networks. They exploit local, positional dependencies in the data. This type of network is used to capture the correlation between adjacent bins in an MD signature.

Residual blocks, as introduced by He et al.^21^ are used to improve accuracy and generality. These blocks contain a so called *skip connection* which allows the model to introduce more or less detail to its feature maps as needed.

This first stage in the network reduces the dimensionality of the input MD signature ${\mathbf {X}}$. This is important as it cuts down on the amount of computations needed for the following GRU layers. As the output of this layer also will be used in the bidirectional branch, the amount of data needed to transfer or store needs to be kept to a minimum.

A residual block in its simplest form is defined as follows, where ${\mathbf {a}}$ is the input to the layer $f$, parametrized by ${\mathbf {W}}$:

7 $$\begin{aligned} {\mathbf {b}} = f({\mathbf {a}}; {\mathbf {W}}) + {\mathbf {a}}. \end{aligned}$$

**Figure 4:**
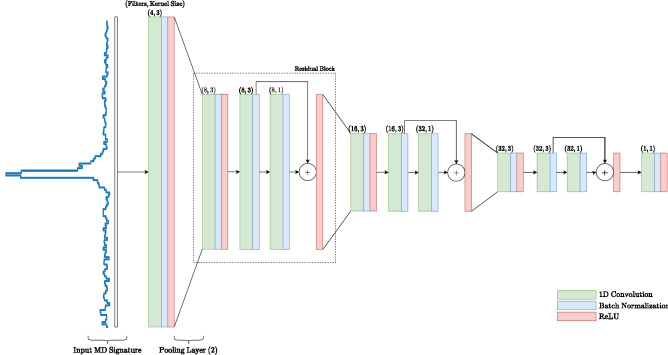
Full network structure of the 1-dimensional convolutional neural network for feature extraction. The input represents an MD signature when considering only a single step in time, noting that an MD signature is a distribution of velocities relative to the radar sensor.

The 1D Convolutional Feature Extractor employed for micro-doppler consists of consecutive residual blocks, followed by a max pooling layer. The residual blocks also make use of Batch Normalization^22^. Eventually, the amount of features is reduced from 128 to 16 values. This makes the network more compact when it reaches the Split BiRNN stage. As a consequence, the amount of data needed for the backward branch for Split BiRNN is reduced, reducing bandwidth needed for the backward branch. This CNN stage is applied to every MD signature *frame* in the input time series, making the CNN a per-frame feature extractor. The structure of this feature extractor stage is shown in Fig. 4.

### Network structure

With both the Split BiRNN and MD signature specific feature extractor introduced, the full model can be constructed for use with Micro-Doppler signatures over time. The network is structured as follows: the MD signatures are fed into the CNN feature extractor introduced in “1D convolutional feature extractor for micro-Doppler signatures” section which reduces the dimensionality of the 128 MD signature bins to 16 features. These features are then fed into the Split BiRNN introduced in “Split BiRNN” section with three GRU blocks^23^ are used as the main recurrent blocks. This amount was chosen empirically, showing dimishing returns when making deeper GRU networks. The GRU recurrent block is chosen for its simpler construction, efficient calculation and similar performance to LSTM.

The amount of inputs needing to be fed to the backward branch are reduced from 128 to 64, consisting of the 16 features from the 1D feature extractor, and 48 features from the intermediate GRU layers. This means if the features were to be sent over a network, the amount of data needed to be transmitted is halved, due to the compression and precalculation performed by the forward branch at the edge.

### Loss function

The loss function used is a variant of Combo Loss^24^, which combines a Dice score and a Cross Entropy to leverage the advantages given by both methods. The dice function handles class imbalance, while the cross entropy is easier to train.

#### Dice loss

The Dice coefficient, or Dice score, is an indicator of overlap commonly used in semantic segmentation in computer vision, where a class is predicted for every pixel in an image^25^. The Dice score ${\mathcal {S}}_{\text {Dice}}$ of a prediction $\hat{\mathbf {y}}$ and its ground truth ${\mathbf {y}}$ is defined as follows. The dice score as used in this work is defined as follows, with $\circ$ denoting the Hadamard or elementwise product:

8 $$\begin{aligned} {\mathcal {S}}_{\text {Dice}} = \frac{2\left\| {\mathbf {y}} \circ \hat{\mathbf {y}} \right\| _1 + 1}{\left\| {\mathbf {y}} \right\| _1 + \left\| \hat{\mathbf {y}} \right\| _1 + 1}. \end{aligned}$$

This value ${\mathcal {S}}_{\text {Dice}}$ is 1 if the prediction and ground truth are exactly equal, and 0 if they differ in every value. The Dice Loss^25^ can then be calculated as

9 $$\begin{aligned} {\mathcal {L}}_{\text {Dice}} = 1 - {\mathcal {S}}_{\text {Dice}} \end{aligned}$$

#### Categorical cross-entropy

Categorical Cross-Entropy (CCE) Loss ${\mathcal {L}}_{\text {CCE}}$ is used with label smoothing. This means that labels with value 1 are replaced by $1-\varepsilon$, and labels with value 0 are replaced with $\frac{\varepsilon }{K-1}$ for a label smoothing hyperparameter $\varepsilon$ and $K$ the amount of classes present in the dataset. The value for $\varepsilon$ chosen here is 0.2. This is to account for the potential misclassification in time, due to potential small inaccuracies in the dataset. There is no exact point at which it can be determined that one activity has started or ended. By utilizing label smoothing a small factor of uncertainty is introduced in the labels.

#### Combo loss

The resulting Combo Loss is the sum of the CCE and Dice Loss, defined as follows:

10 $$\begin{aligned} {\mathcal {L}} = {\mathcal {L}}_{\text {CCE}} + {\mathcal {L}}_{\text {Dice}} \end{aligned}$$

When dealing with imbalanced data, the addition of a Dice Loss term to the Categorical Crossentropy was found to give an improvement.

### Training method

The network defined in “Network structure” section is trained using minibatch stochastic gradient descent. Every batch, snippets of a randomly sampled length $l \in [100, 1000]$ and starting offset $o \in [0, N-l]$ are selected from different recordings in the dataset. By introducing this step, the model can learn to make predictions with arbitrary starting conditions, and the backward branch can handle multiple window lengths. This gives stable inputs to the network and avoids recalibration for new environments and datasets. Both the forward and backward branch are trained simultaneously using the loss introduced in “Combo loss” section. The Adam^26^ optimizer is used with learning rate $10^{-3}$.

### Dataset

#### Radar fundamentals

**Figure 5:**
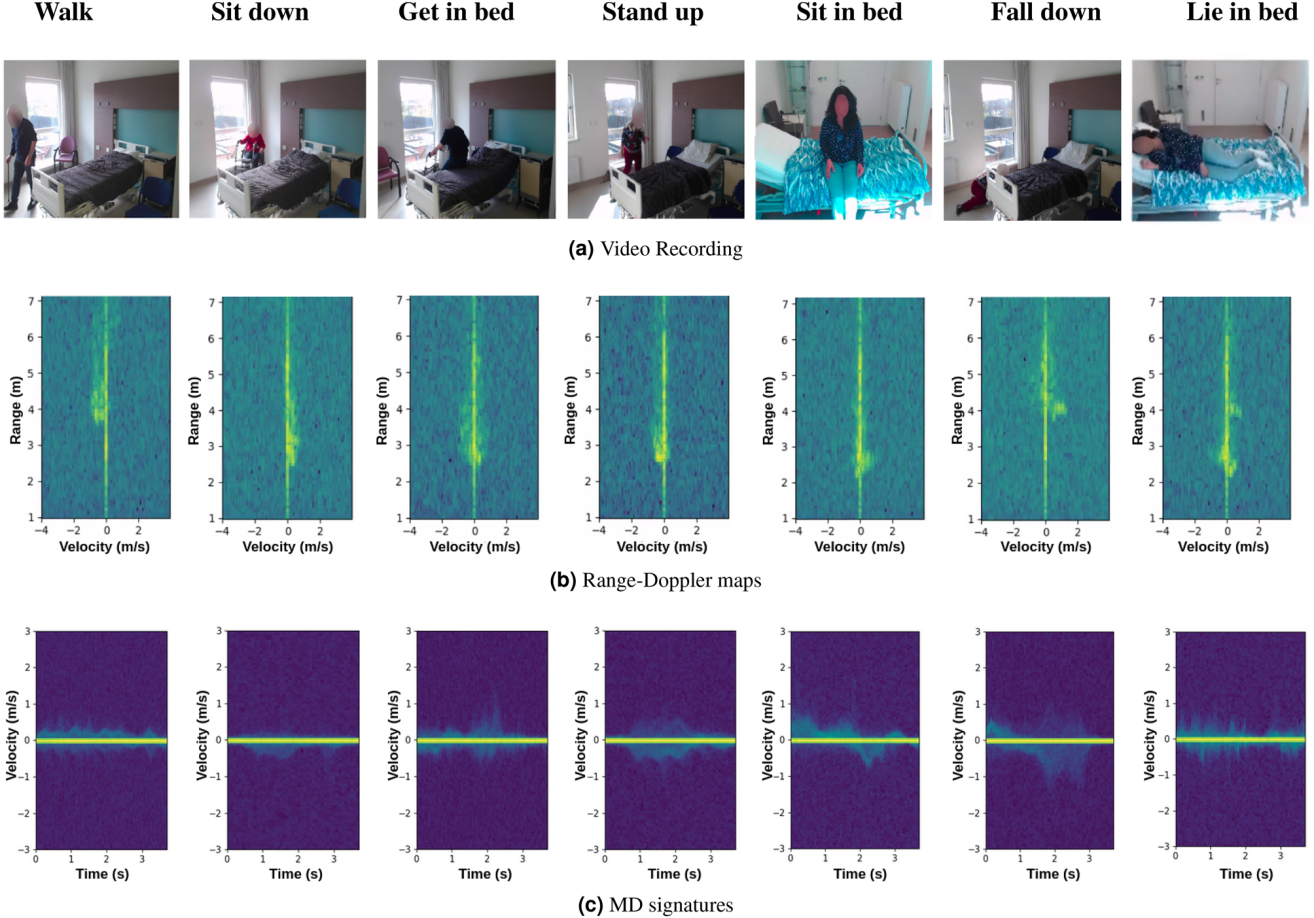
Visualisation of input data in different forms (adapted from Bhavanasi et al.^14^). In (**a**) example activities are seen, (**b**) shows the corresponding Range–Doppler maps in dB, and (**c**) shows the MD signatures in dB.

The Frequency-Modulated Continuous Wave (FMCW) radar is an active sensor that continuously emits electromagnetic signals through transmitting antennas. The signals are reflected by the target and captured by an array of receiving antennas. Essential information about the targets such as range, angle, and speed are then extracted from the reflections based on the time delay or phase shift (i.e., the MD effect^5^). The RD maps, which give information on the range and the velocity of the target, are generated by applying the two-dimensional Short Time Fourier Transform (STFT) on the reflected signals^4^. The discretized velocity ranges is referred to as Doppler bins. An MD signature is obtained from RD maps by summing over the range dimension and concatenating over the time dimension. Figure 5 shows examples of MD signatures and RD maps of different activities.

#### PARRad

The model is trained and evaluated on the PARrad dataset^14^. In total, 22 hours of radar data is present in the dataset, split into two subsets: Homelab and Hospital, both simulating hospital rooms. This work focuses on the Hospital subset contained in PARrad, containing 13359 activities spread over 9 combined classes. The classes available in the dataset are shown in Table 1, with visual examples shown in Fig. 5. The Hospital dataset is comprised of 20 test subjects performing different activities in 4 different 10-min sessions. Two of these sessions are regular, with the test subject performing the tasks as is told. The other two sessions are analogous to the first two, except a cane and walker are used respectively. This is to account for the usage of these walking aids by the elderly population.

**Table 1:** Classes available in PARrad, including the transformations made by combining different classes.

Activity	Merged activity	Samples
Walk to room	Walk	149,880
Walk to chair		
Walk to bed		
Fall on the floor	Fall	33,435
Stand up from the floor	Stand up	99,050
Stand up from chair		
Stand up from bed		
Sit down on chair	Sit down	55,010
Sit down on bed		
Get in bed	Get in bed	29,665
Lie in bed	Lie in bed	23,040
Roll in bed	Roll in bed	30,555
Sit in bed	Sit in bed	21,120
Get out bed	Get out bed	32,225

This dataset contains RD maps and MD signatures from two different radars, a Texas Instruments (TI) xWR14xx radar with center frequency of 77 GHz, and another TI xWR68xx radar with center frequency of 60 GHz. These are placed in different corners of the hospital room, and capture activities simultaneously. This means every capture session is effectively duplicated, albeit with differences in the field of view of the radar sensors.

The captured MD signatures consist of 128 Doppler bins, which were averaged from the 93 range bins in their corresponding RD maps. These MD signatures are stacked over time, so there is a datapoint ${\mathbf {X}} \in {\mathbb {R}}^{t\times 128}$ for every recording in the dataset, where $t$ varies from recording to recording. $t$ is commonly around $t=6666$, which corresponds to 10 min real time due to the frame length being ${0.09}\,{\hbox {s}}$.

The MD signatures made available in the dataset are used for the presented model. The amplitude in MD signatures corresponds to the average velocity of detections. All the individual MD signature frames are standardized in a pre-processing step to have a mean at 0 and standard deviation of 1. This is a common practice when training deep neural networks, because it helps convergence. Standardizing per frame instead of over an entire dataset implies a loss of information about the relative amplitude of one frame to the next. However, the experimental tests have shown that this does not affect the performance of the resulting model.

## Results

This Section is structured as follows: In “Real time inference” section, hop sizes and window lengths of Split BiRNN are tuned for PARRad to minimize the amount of computations, while maximizing the accuracy. In “Comparison to baseline model” section, the Split BiRNN model is tested against an analogous state-of-the-art BiRNN model from^8^ with the optimal hop size and window length for Split BiRNN determined in “Real time inference” section.

### Real time inference

**Figure 6:**
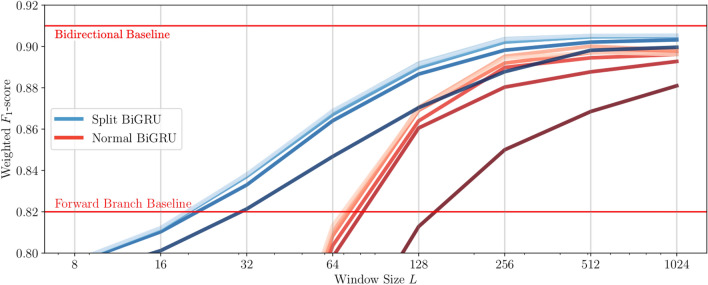
Weighted $F_1$-score comparison versus hop sizes. The gradients correspond to hop sizes $\in \{\frac{L}{1}, \frac{L}{2}, \frac{L}{3}, \frac{L}{4}, \frac{L}{5}, \frac{L}{6}\}$, with darker colours corresponding to a larger hop size *H*. A lower and upper bound are marked in red. These lower and upper bounds correspond to the accuracy achieved using only the forward network, and the full (forward and backward) network on an entire recording. The weighted $F_1$-scores lower than the baseline forward GRU are not shown, as these constitute configurations that are worse than using a regular recurrent neural network.

The technique is tested on window lengths $L \in [8, 1024]$. With a frame time of 90ms, this corresponds to sections of ${0.72}\,{\hbox {s}}$ to ${92.16}\,{\hbox {s}}$. For every window length, the model is tested on 6 hop sizes expressed as fractions from $H = L$ to $H = \frac{L}{6}$. Figure 6 shows the performance of Split BiGRU using a windowed backward branch on a fivefold cross validation compared to that a regular BiGRU with analogous structure to the Split BiGRU.

As expected, a lower hop size corresponds to lower accuracy. Using a hop size of $\frac{L}{3}$, corresponding to an overlap of $\frac{2}{3}$, yields high results, comparable to larger overlaps. Once $L = 256$ (i.e., a duration of 23.04s), a high accuracy is achieved with diminishing returns after this point. This means the optimal predictions for the model can be obtained within $H = \frac{L}{3} = {7.680}\,{\hbox {s}}$ in the PARrad dataset. This is below the accuracy of the full model of 91%, which is expected. However, the lower accuracies for a small window length is unexpected. Ideally, the backward model only adds information leading to a higher accuracy than the forward model. A possible explanation is that averaging is used before the final layer, instead of using an additive technique where the backward branch *corrects* predictions made by the forward branch.

#### Speed of classification

The speed at which inference occurs is vital to the proper functioning of the framework in a real world setting. Time for prediction is used as an approximate metric for computational complexity. Inference is performed using an NVIDIA Geforce RTX 2080 TI GPU and an Intel Xeon Silver 4210 10-core CPU 2.20GHz. The optimal window length of 512 is used for the backward branch and baseline bidirectional model. The results can be seen in Table 2.

**Table 2:** Timing results of the two Split BiGRU branches and a baseline BiGRU.

Model	Frames	Number of model parameters	Execution time (ms)
Forward branch	1	13,350	$1.664 \pm 0.227$
Backward branch	512	6585	$36.645 \pm 3.170$
Bidirectional model	512	21,462	$81.463 \pm 6.516$

The forward step takes approximately 1.7 ms to process a single ${90}\,{\hbox {ms}}$ frame. Reusing calculations from this step in the backward step and performing the backward step takes approximately 36.6ms over a 46.08s timespan, which is an optimal hop size as seen in Fig. 6. A potential speedup is possible by batching multiple calculations.

### Comparison to baseline model

In a real-life, real-time setting, a traditional BiRNN can only be used to predict classes over a sliding window. The highest accuracy using this model is obtained by using a large sliding window, and a short hop size. This means many redundant calculations are performed. In case a large amount of radar streams are processed simultaneously, these computations would put a large burden on the computing unit. If the response to a prediction is time-sensitive, as is the case with fall detection, the hop size would need to be short. The Split BiRNN solves this by storing long-term states in a forward branch, and using intermediate computations in a backward step which reduces bandwidth, while retaining accuracy. As seen in Table 2, a single frame of the MD stream using the forward branch is processed in 1.664ms, and a calculation on 512 frames using the backward branch (using intermediate calculations from the forward branch) requires 36.645ms. The bidirectional network requires 81.463ms to execute on 512 frames. When using this configuration of hop size and window length, a central computing unit needs to only expend 44.98% of the computation time necessary compared to using a regular bidirectional model. The baseline state-of-the-art model used to compare the presented Split BiRNN technique with is a modified 1D-CNN-LSTM, introduced by Vandersmissen et al.^8^ and also employed by Zhu et al.^27^. The original technique presented by Vandersmissen et al. has a different 1D CNN feature extractor, and only features a single LSTM layer. The version employed in the following comparison contains 3 GRU layers instead of a single LSTM layer, and a higher CNN depth. However, fundamentally the structures of the original 1D-CNN-LSTM and the modified version used in this work are very similar, but with different parametrization allowing for a proper comparison between a Split-BiRNN and normal BiRNN.

In Table 3, a baseline BiGRU is compared to the Split BiGRU presented in this work. As expected, the forward branch performs worse than the windowed bidirectional models, with an average weighted $F_1$-score of 0.825. However, once the sliding backward branch is introduced, we see the performance improving to an $F_1$-score of 0.907, surpassing that of the baseline BiGRU, which has an $F_1$-score of 0.895.

**Table 3:** Comparison of weighted $F_1$-scores of the two Split BiGRU branches and a baseline BiGRU.

Model	Weighted $F_1$-score
Baseline bidirectional GRU^8^ (L = 512, H = 171)	0.895
Forward branch	0.825 (− 0.070)
Sliding backward branch (L = 512, H = 171)	**0.907 (+ 0.012)**

The highest score value is indicated in bold.

## Conclusion

A novel method based on Recurrent Neural Networks is proposed to make real-time predictions on streaming data, and leverage the performance of bidirectional RNNs, while keeping the amount of redundant computations to a minimum. This model can be used on a single machine, or split over edge devices and a central server to accommodate for a large amount of different predictions. The forward branch compresses the data needed to be sent to the cloud server by half, reducing the bandwidth needed for the proper functioning of the presented technique. This model can achieve similar results to that of a regular Bidirectional RNN network and is able to be executed efficiently and in real time.

Future research will focus on the investigation of methods to tune the window length needed to make a backward prediction. In cases where an activity takes a long time, there might not be an added value to include the windowed backward branch. By leveraging uncertainty in the forward branch to guess which predictions need to be processed further using a backward branch, the hop size and window length could be chosen dynamically. This has the potential advantage of reducing redundant computations and improving accuracy. Investigating the impact of data loss is also worthwhile, as this model does not take packet loss into account, which may occur in the network.

## Data Availability

The dataset (PARRad) analysed during the current study are at the time of writing not publicly available due to its corresponding accepted manuscript^14^ but are available from https://sumo.intec.ugent.be/radar.
